# Product development and X-Ray microtomography of a traditional white pan bread from plasma functionalized flour

**DOI:** 10.1016/j.lwt.2022.114326

**Published:** 2023-01-15

**Authors:** Sonal Chaple, Chaitanya Sarangapani, Shannon Dickson, Paula Bourke

**Affiliations:** a School of Biosystems and Food Engineering, University College Dublin, Belfield, Dublin 4, Ireland; b School of Food Science and Environmental Health, Technological University Dublin, Dublin 7, Ireland; c School of Culinary Arts and Food Technology, Technological University Dublin, Dublin 7, Ireland

**Keywords:** Pan bread, Cold plasma processing, Wheat flour, Farinograph analysis, X-ray microtomography

## Abstract

Cold plasma (CP) technology has emerged as a novel non-thermal technology with the potential to improve food quality or impart functionality to ingredients. Our previous studies on wheat flour demonstrated how the structure and functionality of wheat flour might be modified using CP to provide an alternative to chemical additives (Chaple et al., 2020). However, understanding of the further effects of plasma functionalized ingredients in existing or new product formulation is limited. This study investigated the effects of CP treatment of wheat flour on traditional white pan bread development. The bread was formulated using plasma functionalized flour (PFF), and critical product characteristic responses were analyzed. Plasma treatment of flour positively affected the bread's expansion ratio, crust color, and water activity. Farinograph analysis suggests improvement in water absorption capacity, dough development time, and dough stability. X-Ray Microtomography (XRMT) analysis was conducted to understand how plasma functionalising the flour impacted the microstructure of bread. The 3D scans suggested no macro-change in the bread matrix compared to control; however, the porosity decreased in line with the increasing plasma treatment duration of the flour. The texture profile analysis showed an improvement in the gluten network developed in the dough developed from PFF. Sensory analysis results showed overall acceptance for bread formulated with PFF compared with a commercial sample. Overall, CP treatment of the flour improved the functionality in relation to dough and bread preparation and can thus provide an alternative to chemical additives in bread making. The CP processes may be modulated to deliver tailored effects for bread product development.

## Highlights

• The expansion ratio of bread improved using plasma functionalized flour.
• The porosity distribution ratio within the bread loaf increased with the flour treatment time.
• XRMT images indicated the increasing porosity distribution in bread with increasing flour treatment duration.
• Sensory analysis showed acceptance of bread developed from plasma functionalized flour using a consumer acceptance test.

## Graphical abstract

**Figure:**
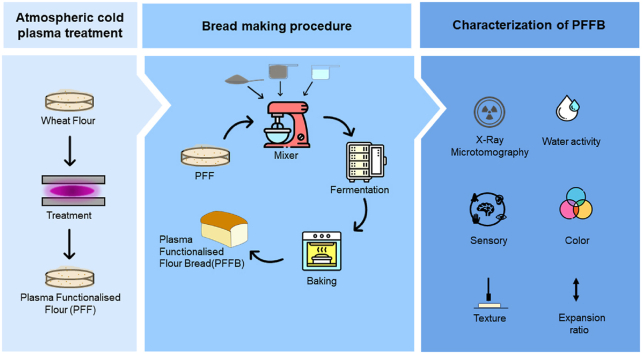

## 1 Introduction

Cold plasma (CP) is a novel non-thermal technology emerging as a flexible and tunable approach to improving the quality characteristics of diverse food systems. Plasma is the fourth state of matter containing quasi-neutral ionized gas, electrons, ions, atoms, UV photons, and charged particles (Pankaj & Keener, 2017). Dielectric Barrier Discharge (DBD) is one of the most widely used plasma configurations in development for food application and can be operated in a batch or continuous mode and provide useful surface exposure. However, a thorough understanding of the plasma system, the process factors, and their interactions with the food system or target under treatment is required to address unmet quality or safety needs.

In the bread-making process, apart from using main ingredients like wheat flour, fat, yeast, salt, and water, there is a common practice to use food additives and processing aids such as oxidants, reductants, enzymes, dough conditioners, etc. These substances are added to improve different properties of bread-like texture, volume, flavor enhancement or moisture retention, etc. (Gioia et al., 2017). However, with an increase in consumers awareness regarding the use of chemical additives and clean-label food products, the food industry is seeking alternatives to the current process to improve the properties of bread, resulting in the reduction/removal of these chemical additives (Asioli et al., 2017; Barros et al., 2021).

Substitution of chemical additives with clean alternatives has been previously explored. For example, the addition of lemon juice as an alternative to chemical analogs due to its acidification properties was studied by (Scarton et al., 2021). Though the addition of lemon juice improved the shelf life of bread, it increased the dough mixing time, produced lower bread volume, firmer crumb with lighter crust. In another study, selected fermentates were studied as a substitute for propionate in bread. The author concluded that fermentates could mimic the antifungal properties of calcium propionate with acceptable sensorial properties at lower concentrations (Samapundo et al., 2017).

The application of a non-thermal technology to improve the properties of bread was proposed previously. The effect of irradiation doses (0.5, 1.0, and 2.0 kGy) on wheat grain and, later, French bread developed from the irradiated flour was investigated. A decrease in falling number was associated with increasing irradiation dose, which was attributed to a change in starch gelatinization properties. However, no change in dough texture, dough development, or dough's gas retention capacity was observed following irradiation (Singer et al., 2006). Misra et al. (2015) treated wheat flour with DBD plasma at 60 and 70 kV for 5 and 10 min and observed the formation of disulfide linkages leading to an improvement in dough strength and mixing time and an increase in viscoelastic properties of strong flour. Bahrami et al., 2016 reported similar results when bread-type wheat flour was treated with air plasma (60 and 120 s at applied power of 40 ± 1 W and 90 ± 1 W). When both soft and hard wheat flour was treated with radiofrequency generated cold plasma, farinograph properties like dough development time and stability time decreased for hard wheat flour but increased for soft wheat flour (Held et al., 2019). Another study noted an increase in specific loaf volume, total loaf volume, and shape formation ratio when flour was treated for 30 or 45 min using gas plasma (Menkovska et al., 2014).

Despite the increased interest in alternative approaches to improve functional properties of grain flours, there is a need to understand the effects of CP treatments on the functional properties and the subsequent behaviour in standard product formulations. Other non-thermal technologies including high-pressure processing, ultrasound, and ozone have been evaluated for use with cereal-based products like bread, cookie dough, or pregelatinized corn flour bread (Bárcenas et al., 2010; Aguirre et al., 2018; Jalali et al., 2020; Carballo Pérez et al., 2018; Safonova et al., 2011).

To the best of our knowledge, there has been no scientific study reported to date on the application of cold plasma for cereal-based product development and their quality factors assessment. Among cereal-based products, bread has been a subject of many scientific studies due to its frequent consumption and essential role in daily diets. Our previous studies on wheat grains and flour identified CP processes for cereal grains decontamination, enhancement of quality factors such as seed germination (Los et al., 2017, 2018, 2019), and pesticide degradation (Sarangapani et al., 2016). Los et al. (2019) observed a decrease in the water contact angle of wheat grain following direct treatment, indicating an increase in the water permeability of grain, but the morphological analysis suggested no impact of plasma on the external surface of the grain.

The plasma process development conducted on wheat flour and grain showed an increase in the water hydration properties and the whiteness index of flour, accompanied by a change in starch crystallinity observed after CP treatment (Chaple et al., 2020). The physical, textural, and sensory properties of cereal-based products depend on both micro and macro-structure, and to develop a product with the desired quality, and it is essential to characterize the microstructure. The objective of this work was to investigate the potential of CP functionalized flour in bread product development. For this purpose, a traditional pan bread, with a well-understood process, was prepared with plasma functionalized flour and compared with control (untreated flour). The effect on critical loaf quality characteristics, sensory acceptability as well as microstructural properties of the bread were analyzed as a function of the plasma process applied to the raw ingredient of wheat flour.

## 2 Materials and methods

### 2.1 Materials

A commercial hard wheat flour (Neill's Spring, Dublin, Ireland) was used in this study. Salt and compressed baker's fresh yeast were obtained from local retailers (Dublin, Ireland). All experimental chemicals and reagents used were of analytical grade and were procured from Merck Dublin, Ireland.

### 2.2 Methods

#### 2.2.1 Cold plasma treatment of wheat flour

**Fig. 1:**
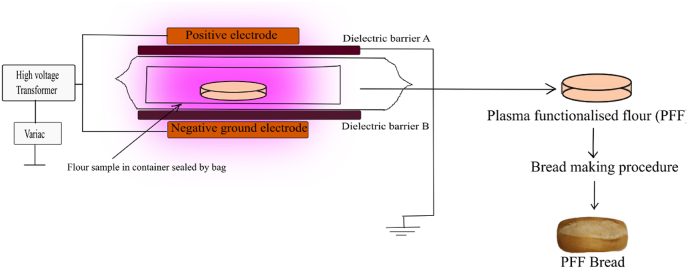
Schematic of the experimental set-up for generation of plasma using dielectric barrier discharge model.

The schematic of the contained high voltage atmospheric air plasma reactor based on a dielectric barrier discharge (DBD) is shown in Fig. 1. This device is described in detail in Sarangapani et al. (2017) and is characterized in (Moiseev et al., 2014; Patil et al., 2014; Han et al., 2016). Wheat flour (15 g quantities) was weighed in a petri dish and placed inside the polypropylene box. The polypropylene box was sealed with packaging film (Cryovac, Ireland). The box was placed between the two circular aluminum electrodes with an outer diameter of 158 mm (Phenix Technologies Inc., MD, USA), and an applied voltage was provided by a step-up transformer (Phenix Technologies, Inc., MD, USA) with an input of 230 V, 50 Hz from the mains supply. All samples were treated at an output voltage of 80 kV RMS over a treatment duration of 10–30 min. All samples were stored for 24 h at room temperature prior to use. The system configuration and characterization of this DBD set-up were previously reported in (Conway et al., 2016; He et al., 2020; Misra et al., 2014; Moiseev et al., 2014; Patil et al., 2014; Sarangapani et al., 2016, 2017).

#### 2.2.2 Bread formulation and bread-making procedure

**Fig. 2:**
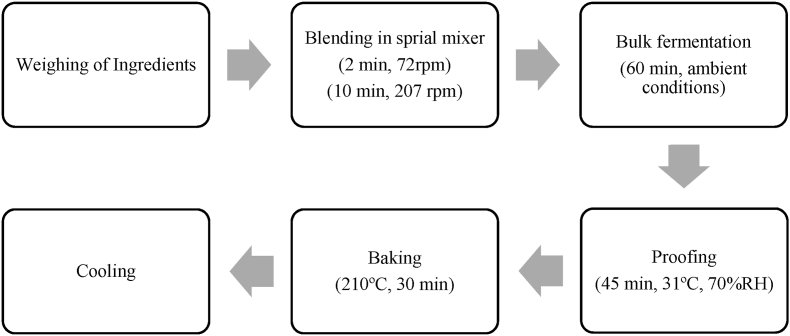
Flow diagram for bread-making procedure of traditional pan bread.

The production of the control bread was based on a straight dough system, as explained in Fig. 2. First, the raw materials [300 g strong flour, 6 g of table salt, 6 g of fat (unsalted butter), 10 g of fresh yeast (AB Mauri Pinnacle Bakers), and 175 g of water] were added to a professional 5 L spiral mixer (Bear Varimixer Teddy, UK) with a stainless-steel dough hook. Next, the control dough was mixed for 2 min at slow speed (72 rpm) to combine the raw materials followed by 8–10 min on high speed (207 rpm) to obtain the optimum temperature of 26 °C. The plasma functionalized flour loaf was processed in the same manner, with the addition of a further 10 g of water (185 g total) to reach an optimum dough temperature of 26 °C.

After the doughs were mixed to the correct temperature and consistency, they were covered to ferment at ambient conditions for 60 min, and the dough was punched down after 45 min. The fermented dough (497 g batches) was then knuckled out into a pad, the ends of the dough were folded to form a rectangle, the dough was rolled up and placed into the rectangular moulds (9cm × 10cm × 17 cm) and fermented again in the proving chamber (M54, Panimatic, France) for 45 min at a temperature of 31 °C and 70% RH. Before the dough was baked, it was scored down the middle and placed into a pre-heated deck oven (Sveba Dahlen, Sweden) of 230 °C, that dropped to 210 °C with the heat settings at 6 (front heat), 4 (top heat), and 2 (bottom heat) for 35 min with 10 s of steam to develop the caramelization of the crust. Finally, the bread was removed from the oven then cooled at ambient bakery conditions. Henceforth, the bread formulated from plasma functionalized flour for 10, 20, and 30 min will be termed PFFB-10, PFFB-20, and PFFB-30. The control was bread formed from untreated flour.

#### 2.2.3 Proximate analysis

The control and plasma-treated flour moisture content were determined by the oven-drying method at 105 °C, and ash content was estimated in a muffle furnace at 500 °C. The protein content of flour was determined by the Kjeldahl method and was calculated by multiplying nitrogen content with a protein conversion factor of 6.25. The fat content was determined by Soxhlet extraction with hexane for 6 h. Finally, carbohydrate content was calculated by the difference method as Total carbohydrate = (100-protein-ash-fat-moisture) (AACC, 2000).

#### 2.2.4 Color analysis

Color has been determined by CIELAB hunter lab colorimeter (Stotto Hunterlab, UK) at room temperature (21 ± 1 °C). The observation angle was 10°, equal to the perception of a human observer, and illuminant D65 was used (daylight source), following the CIE recommendations. For calibration, a white (X-78.8, Y-83.5, Z-89.8) and black reference standard were used before the measurements. A 2.5-inch glass sample measuring cup was filled with crushed bread crumb/crust, and the color was measured. Color parameters obtained were L* (L* = 0 [black] and L* = 100 [white]), a* (–a* = greenness and +a* = redness) and b* (–b* = blueness and +b* = yellowness).

#### 2.2.5 Expansion ratio

The expansion ratio is a measure of dough's rise during the fermentation period. The expansion ratio is calculated for dough after the mixing stage as per the method described by (Besbes et al., 2013). Equal pieces of dough were placed at the bottom of the cylindrical flask, and its vertical expansion was measured throughout fermentation up to the proofing stage. The formula for the calculation of the expansion ratio is in equation (1).

Eq (1) $$Expansion\;Ratio=\frac{Final\;rise\;of\;the\;dough}{Initial\;height\;of\;the\;dough}$$

#### 2.2.6 Water activity

Water activity is a measure of the ability of the sample to retain water and is a measure of the product's shelf life. Water activity indicates how tightly water is bound structurally or chemically with a given substance. It is the ratio of the sample's vapor pressure to the water's vapor pressure at a given temperature and relative humidity. The water activity of bread was measured using Aqua Lab Series 3 (Lennox, USA). The instrument was standardized using 6.00 mol/kg of NaCl in H_2_O, which gives 0.760 value at 25 °C. Approximately 2 g of the sample was placed in the water activity container and the water activity meter chamber.

#### 2.2.7 Farinograph analysis

The dough mixing properties were carried out with Brabender Farinograph (Dublin, Ireland) according to the method of AACC approved method 54-21 (AACC, 2000). The 300-mixing bowl was used with a standard operating speed of 63 rpm. The mixing bowl was temperature-controlled using a thermostat at 30 °C throughout the experiment. The dough was added to the mixing bowl and dry mixed for 1 min, followed by the addition of water at 30 °C through a large burette. The test dough was mixed for 35 min, and the following parameters were estimated: farinograph water absorption (14% mb); farinograph stability time (the difference in the time when it first reaches 500 FU line to when it first exits 500 FU line); dough development time (time required to achieve optimum dough development); mixing tolerance index (the difference between the tip of the curve at the peak and the top of the angle measured 5 min later, FU); degree of softening (the difference between the center of the graph at maximum resistance to mixing and the center of the chart at a point 12 min later, FU).

#### 2.2.8 XRMT analysis

X-ray microtomography (XRMT) has been applied to study food microstructure such as air bubbles or cells, starch granules, protein assemblies, and food biopolymer matrices (Cafarelli et al., 2014b; Laverse et al., 2012). XRMT exploits differences in attenuation of X-ray radiation by material to generate transmission images of samples. A 3-D tomograph is reconstructed from multiple images taken from different angles of the sample (Devahastin, 2017). In this study, the sample image acquisition was carried out using a VTOMEX L 300 system (Waygate Technologies, Baker Hughes Digital Solutions GmbH, Germany), described in detail in Kruth et al. (2011). Each sample was mounted on the rotation stage and exposed to X-ray radiation for image capture. The scans are completed at the voltage of 180 kV voltage, a current of 80 μA, and an integration time of 250 ms. A 300 kV open X-ray source was used, giving 14.95 μm focal spot onto a tungsten transmission target. The bread sample was rotated 360° during scanning. The distance between the focus detector and the focus object is 840 mm and 42 mm, respectively. A 400 by 400 mm screen with 2000 × 2000 pixels is utilized with a GE DXR250 digital detector array (Camarada et al., 2018).

The scanned images of the bread samples were exported, and the results were quantified using myVGL 3.2.5 64 bit volume graphics software (https://www.volumegraphics.com/en/products/myvgl.html). All images were reduced to 16-bit unsigned size with two intensity ranges. Approximately 300 scans were segmented to obtain similar-sized rectangular (73.115 × 36.55 × 73.115 mm) regions of interest from the center part of the sample to provide uniformity. The reconstructed microtomographic images were viewed at nominal resolution 73 μm, 2.7x magnification, and 6000 resolution along each edge of the voxels in the 3D arrays—voxel size for all images was 0.0731891 × 0.0732624 × 0.0731891 mm. Porosity was calculated as the percentage of voxels designated as pores divided by the total volume of interest (Demirkesen et al., 2014).

#### 2.2.9 Texture analysis

Texture analysis of bread was conducted according to AACC method 74.09 (AACC, 1999) of fresh bread using texture analyzer TA. hD*plusC* with TPA Exponent Software (Stable Micron Systems, Mason Technology, Ireland) with 5 kg load cell. The middle bread slices of 25 mm thickness underwent a compression cycle up to 40% deformation of their original height with a 35 mm flat end cylindrical aluminum probe (P/35). The compression test was performed using the selected settings of 1 mm s^−1^ pre-test, 1.70 mm s^−1^ test speed, and 10 mm s^−1^ post-test speed with 5 g trigger load and 40% strain. Five replicates were analyzed for each sample, and hardness in force (N) was estimated.

#### 2.2.10 Sensory analysis

The sensory analysis test was conducted using the 9-point hedonic scale on plasma-treated, control, and commercial samples. The samples were served to 20 semi-trained panelists from a population of staff and students at Technological University Dublin ranging from age 20–35 years of age. A 9-point hedonic scale was estimated to measure participant's preference of the samples. The samples were coded randomly, presented in identical containers, and served to panelists for evaluation. The panelists were given categories converted to numerical scores ranging from 1 to 9, with 1 representing dislike extremely and 9 as like extremely (Iwe, 2002). The sensory analysis of the following attributes was performed: appearance, crust color, crumb color, aroma (crust), aroma (crumb), texture (by fingers), and overall acceptance of bread.

#### 2.2.11 Statistical analysis

Statistical analysis was performed using SPSS software (IBM statistical analysis version 25.0). The results were analyzed using one-way ANOVA. The significance among the samples was compared at p ≤ 0.05 by Tukey HSD test post-hoc comparison. All the tests were performed in triplicate, and the average of the tests are presented.

## 3 Results and discussions

### 3.1 Bread formulation and expansion ratio

**Fig. 3:**
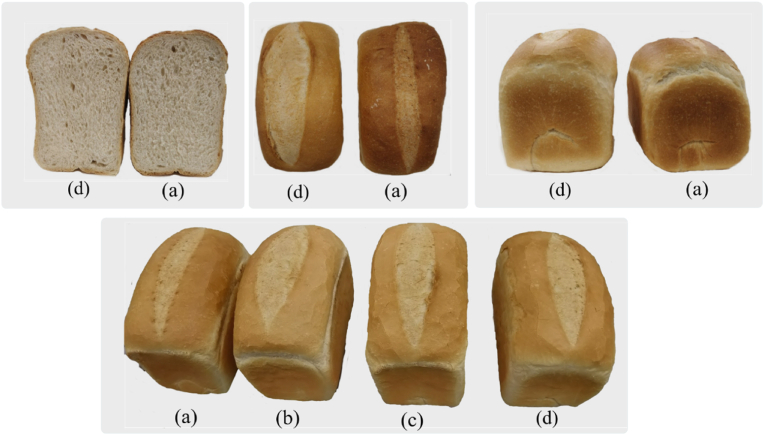
Images of bread from cross sectional, top and rear view, where (a) control is bread formulated from untreated flour (b) PFFB-10 is bread formulated from flour treated for 10 min (c) PFFB-20 is bread formulated from flour treated for 20 min (d) PFFB-30 is bread formulated from flour treated for 30 min.

The bread was formulated from each of the control and plasma functionalized flours. The plasma-treated flour behaved similarly to the control during the whole bread development process, as shown in Fig. 2. The cross-sectional, rear, and front images of the control and PFFB are shown in Fig. 3. In bread-making, the fermentation or proofing process aims to increase dough volume and generate small metabolites, which will contribute to the flavor and taste profile of bread. Due to yeast activity, sugars are converted into carbon dioxide and alcohol during fermentation, and short-chain fatty acids are released due to the action of lactic acid bacteria. The carbon dioxide formed during this process aids dough expansion, leading to a volume increase of dough, which ultimately provides crumb structure (Rosell, 2011). The expanding gas cells in the loaf are surrounded by a gas-liquid interface stabilized by the gluten matrix and the adsorbed surfactants such as polar lipids gliadins. The dough's gas retention capacity improves with more stable gas cells leading to an improved dough expansion (Sang et al., 2020). A significant increase in the expansion ratio of dough was observed with plasma treatment (p < 0.05). The expansion ratio of dough formed from flour treated for 30 min was 2.86 ± 0.016 while for control was 2.73 ± 0.024. After baking, the PFFB-30 loaf height was increased by 6.25 ± 0.55% compared to the control. Fig. 3 shows the images of control bread and PFFB from different angles (cross-section, rear, and front). It can be observed that PFFB-30 bread has a higher height than control. Plasma treatment of the flour has affected the formation of the dough matrix resulting in the bread to rise higher.

The increase in expansion ratio can be related to the improvement in the gluten structure of flour and hydration properties. Many authors noted the improvement in flour hydration and hydration capacity due to plasma treatment (Chaple et al., 2020; Sarangapani et al., 2015; Thirumdas et al., 2016, 2017). This increase in water hydration properties was attributed to increased capillary action and surface energy, ultimately leading to increased water uptake of plasma functionalized flour (Grzegorzewski et al., 2010; Thirumdas et al., 2016). Previously, Held et al. (2019) reported that radiofrequency plasma treatment improved the viscoelastic gluten network of hard wheat flour. Misra et al. (2015) also observed an improvement in dough development time (peak time) and strength of the dough (peak integrals), attributed to disulfide bond formation after 60 kV plasma treatment. In addition, ozone formed during the plasma treatment acts as an oxidizing agent, leading to disulfide bonds between cysteine moieties.

Industrially, many leavening agents are added to achieve this desirable dough expansion: sodium bicarbonate, potassium bicarbonate, and ammonium bicarbonate (De Leyn, 2014). The bicarbonate reacts with water during the mixing and proofing stage to produce CO_2_ and promote bread volume rise (Miś et al., 2016). The addition of oxidizing agents such as potassium bromate to remove sulfhydryl groups to promote the formation of disulfide bonds is a common practice (Shanmugavel et al., 2020). Thus, CP treatment of flour could be exploited as an alternative to the addition of these chemical agents.

### 3.2 Proximate analysis

The proximate analysis of control and plasma-treated flours is shown in Table 1. The moisture content of control and plasma-treated flour samples was in the range of 11.66%–13.37%. The sample's moisture content did not significantly change due to in package cold plasma treatment. Other studies that employed a similar in-package DBD set up reported no change in moisture content for cheese samples using dry air or MA65 (65%O_2_, 30% CO_2_, 5%N_2_) (Wan et al., 2021). In contrast, a reduction in moisture content was reported in xanthan gum (discharge gas: air, 50–60 W power), long-grain brown rice (discharge gas: air, 1–3 kV voltage), brown rice (discharge gas: air, 1–3 kV voltage), and basmati rice (discharge gas: air; 30–40 kV) when treated with low-pressure plasma (Bulbul et al., 2019; Chen, 2014; Chen et al., 2012; Thirumdas et al., 2016). A decrease in moisture content can happen due to the breakdown of water molecules into oxygen radicals.

**Table 1:** Proximate analysis of wheat flour samples. The control is untreated flour, Flour-10 min, Flour-20 min and Flour-30 min are flour samples treated for 10, 20 and 30 min respectively. All the data are expressed as mean ± standard deviations. Means with different superscript letters in a column differ significantly (*p* < 0.05).

Samples	Moisture (%)	Carbohydrates (%)	Fat (%)	Protein (%)	Ash (%)
**Control**	13.376 ± 1.61^a^	77.555 ± 1.28^a^	0.981 ± 0.006^a^	7.280 ± 0.15^a^	0.806 ± 0.16^a^
**Flour-10 min**	12.069 ± 0.06^a^	78.977 ± 0.23^a^	0.976 ± 0.012^a^	7.015 ± 0.1^a^	0.960 ± 0.04^a^
**Flour-20 min**	12.313 ± 0.04^a^	79.191 ± 0.48^a^	0.983 ± 0.005^a^	6.536 ± 0.47^a^	0.975 ± 0.04^a^
**Flour-30 min**	11.666 ± 0.77^a^	79.467 ± 1.04^a^	0.967 ± 0.01^a^	7.015 ± 0.31^a^	0.884 ± 0.77^a^

The protein content of the samples was in the range of 6.53–7.2%, while the ash content was in the range of 0.806–0.975%, and the fat value was in the range of 0.976–0.983%. No change in carbohydrate, protein, fat, and ash content of both control and plasma-treated wheat flour was observed. Thus, this plasma treatment did not affect the macro-nutritional properties of wheat flour.

### 3.3 Color

Table 2 provides the data on the effect of plasma treatment on the color profile (L*, a*, b*) of the crust and crumb. The color value of the sample was corelated by treatment time duration. For a* value, although the values varied, no significant differences were observed between the control and treated crust and crumb values. The L* value of crust was significantly different (p < 0.05) among control and treated flour. The L* value of control crust was 47.60 ± 0.64 and for PFFB-30 is 55.70 ± 0.58. The application of plasma treatment increases the L* value significantly (p < 0.05), signifying a bleaching effect of the plasma-generated species. The L* value of crumb did not show much significant difference. The b* value for crust PFFB-30 (37.10 ± 0.32) was significantly higher (p < 0.05) than control (32.20 ± 1.46).

**Table 2:** Color analysis of bread samples. The control is untreated flour bread, PFFB-10, PFFB-20, and PFFB-30 are bread formulated from flour treated for 10, 20 and 30 min respectively. All the data are expressed as mean ± standard deviations. Means with different superscript letters in a column differ significantly (*p* < 0.05).

Sample	Crust			Crumb		
	L*	a*	b*	L*	a*	b*
**Control**	47.60 ± 0.64^a^	17.05 ± 0.49^a^	32.20 ± 1.46^a^	72.06 ± 0.39^a^	0.94 ± 0.03 ^a^	15.37 ± 0.18 ^a^
**PFFB-10**	49.63 ± 0.42^b^	17.2 ± 0.27^a^	33.45 ± 0.17^ab^	72.07 ± 0.42^a^	0.93 ± 0.04^a^	16.13 ± 0.14^a^
**PFFB-20**	51.16 ± 0.12^b^	16.80 ± 0.19^a^	34.88 ± 0.07^bc^	71.88 ± 0.08^a^	1.016 ± 0.01^a^	17.01 ± 0.05^b^
**PFFB-30**	55.70 ± 0.58^c^	16.35 ± 0.07^a^	37.10 ± 0.32^c^	71.86 ± 0.15^a^	1.02 ± 0.014^a^	17.34 ± 0.01^c^

The color of the bread crust treated with plasma treatment after 5 min also showed a change in color, suggesting the oxidation of reducing sugars by the reactive oxygen species, thus preventing the Maillard reaction between glucose and amino acids which causes browning of bread. A similar increase in the lightness value of bread was reported for ozonated flour bread (Obadi et al., 2018; Sandhu et al., 2011). Wheat flour contains carotenoid pigments, where the double bonds are susceptible to the reactive oxygen species. In the aleurone layer of a wheat kernel, a polyphenol oxidase enzyme is present, which plays a significant role in darkening wheat products (Bhattacharya et al., 1999). Previous studies have reported that plasma treatment inactivated polyphenol oxidase in potatoes and cloudy apple juice (Illera et al., 2019; Kang et al., 2019).

### 3.4 Water activity

**Fig. 4:**
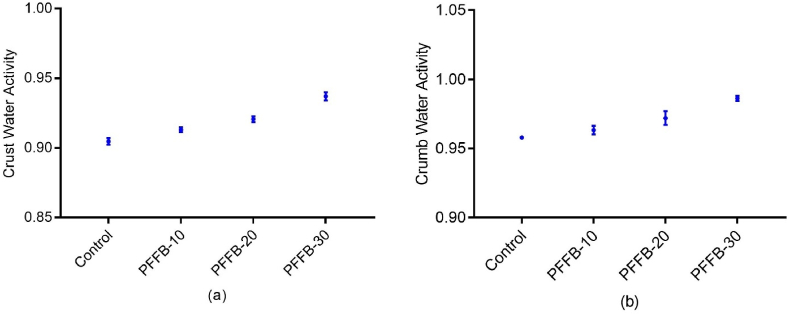
Water activity of bread samples where (a) is crust water activity and (b) is crumb water activity for different samples. The control is untreated flour bread, PFFB-10, PFFB-20 and PFFB-30 are bread formulated from flour treated for 10, 20 and 30 min respectively. All the data are expressed as mean± standard deviations. Means with different superscript letters in a column differ significantly (*p* < *0.05*).

The water activity value for both crust and crumb of bread samples are shown in Fig. 4. The water activity of the control crumb (0.958 ± 0.0816) was similar to that of PFFB-30 (0.974 ± 0.017), suggesting no significant changes in the water activity of the crumb. However, the water activity of crust PFFB-30 (0.931 ± 0.002) was significantly higher (p < 0.05) than that of control (0.904 ± 0.002). Our previous work showed an improvement in the water holding capacity for 30 min plasma-treated wheat flour (2.87 ± 0.16 g/g) compared to control (2.07 ± 0.04 g/g) (Chaple et al., 2020). Held et al. (2019) also reported an increase in water absorption capacity attributed to starch modification in soft wheat and hard wheat flour. Thus, the plasma treatment can improve the water intake capacity of the flour, providing moist bread.

Based on the color analysis, control bread was darker in color than plasma-treated bread, suggesting the possibility of the Maillard reaction. The Maillard reaction occurs in three stages; in the first stage, the carbonyl group on a sugar reacts with a protein or amino acids producing glycosylamine and water, the glycosylamine compound further isomerises to give ketosamine by amodari rearrangement. In the third and final stage, the ketosamine reacts in different steps giving a brown-colored complex compound (Çelik & Gökmen, 2020). In the control sample, the Maillard reaction led to water molecules' formation, which may evaporate due to the high oven temperatures (approx 230 °C) during baking, resulting in reduced water activity.

### 3.5 Farinograph properties

The farinograph is used to assess the baking quality and performance in plasticity and mobility of wheat doughs and records the behaviour of dough against the mixing action of blades over time and at a specific constant speed and temperature (Locken et al., 1972). The results of farinograph studies for untreated and plasma-treated flour are summarized in Table 3. Water absorption capacity (corrected to 14% mb) is the amount of water needed to balance the farinograph curve to specified maximum torque (500-FU line). The water absorption of control, 10 min, 20 min, and 30 min dough were 63.25 ± 0.95%, 66.25 ± 0.95%, 66.15 ± 0.45%, and 70 ± 0.6%, respectively. The water absorption of the flour increased significantly (p < 0.05) after 30 min of treatment time. The major factors influencing the water absorption properties are the amount of starch damage and gluten-forming proteins (Vizitiu & Danciu, 2011). Various authors have reported modification of starch by cold plasma treatment (Lii et al., 2002a; 2002b; Wongsagonsup et al., 2014; Zhang et al., 2014). Reactive species may induce depolymerization, cross-linking, functionalization on the starch molecule modifying starch properties (Zhu, 2017). Our previous work also suggested that plasma-treated flour possess higher water hydration capacity, high peak gelatinization temperature, and changes in crystallinity when compared to control (Chaple et al., 2020). This increase in water absorption capacity may correlate with the required addition of an extra 10 g of water during the bread-making process.

**Table 3:** Farinograph properties of wheat flour. The control is untreated flour, Flour-10 min, Flour-20 min and Flour-30 min are flour samples treated for 10, 20 and 30 min respectively. All the data are expressed as mean ± standard deviations. Means with different superscript letters in a column differ significantly (*p* < 0.05).

Farinograph properties	Water absorption (corrected to 14% mb)	Development Time (min)	Stability (min)	Degree of softening (FU)	Farinograph Quality Number
**Control**	63.25 ± 0.95^a^	1.5 ± 0.45^a^	1.1 ± 0.1^a^	86.5 ± 4.5^a^	15±0^a^
**Flour-10 min**	66.25 ± 0.95^a^	2.1 ± 0.45^b^	1.7 ± 0.1^ab^	89 ± 11^a^	22±1^b^
**Flour-20 min**	86.5 ± 4.5^a^	2.1 ± 0.55^b^	2.15 ± 0.15^b^	77±5^a^	21.5 ± 0.5^b^
**Flour-30 min**	70 ± 0.6^b^	3.1 ± 0.3^c^	2.05 ± 0.05^b^	89.5 ± 2.5^a^	31±1^c^

The dough development time is the time required for the top of the curve to reach 500 FU line. It refers to the initial water addition and the point at which the dough seems to have the best elastic and viscous qualities for gas retention (Vizitiu & Danciu, 2011) and is also referred to as peak time in the mixolab. The control dough development time was 1.5 ± 0.45 min, which increased to 3.1 ± 0.3 min for dough made with 30 min plasma-treated flour. The dough development time is closely related to changes in the gluten structure during the early phase of dough mixing. It also depicts the elasticity of the dough; the better elasticity of the dough, the longer the development time (Locken et al., 1972). The disulfide bonding between proteins is a key factor for dough properties, and cold plasma has been reported to enhance the dough properties by the oxidation of sulfhydryl groups of disulfide bonds (Xia & Cooks, 2010). Misra et al. (2015) reported an increase in the optimum mixing time when both strong and weak wheat flour was exposed to cold plasma treatment (up to 70 kV for 10 min). The dough strength was also reported to improve with air plasma (Bahrami et al., 2016; Misra et al., 2015). However, in contrast, a decrease in dough development time was observed when hard winter wheat was subjected to radiofrequency cold plasma, while an increase in dough development time was observed for soft wheat flour (Held et al., 2019).

The dough stability represents the time during which the maximum dough consistency does not change significantly, and it is an indication of flour's tolerance to mixing (Li et al., 2019). The dough stability value of control, 10 min, 20 min, and 30 min dough are 1.1 ± 0.1min, 1.7 ± 0.1 min, 2.15 ± 0.15 min, and 2.05 ± 0.05 min, respectively. The plasma treatment increased the dough stability as a function of treatment duration and was significantly more stable (p < 0.05) after 20 min of treatment.

The degree of softening measures the dough resistance to mechanical stirring. The cold plasma treatments did not significantly affect the degree of softening of the dough. A high water absorption capacity with a lower degree of softening indicates good quality flour (Aydoğan et al., 2015). A decrease in the degree of softening after 30 and 45 min of cold plasma treatment was reported on wheat flour (Menkovska et al., 2014).

Farinograph Quality Number was also significantly (p < 0.05) improved with the application of plasma treatment. Overall, plasma treatment improved farinograph dough quality by affecting the behaviour of protein and starch fractions in wheat flour.

### 3.6 XRMT analysis

Food microstructure is defined as the study of the spatial arrangement of its structural arrangement and their interactions (Morris & Groves, 2013). Food microstructure is essential to understand its physical behaviour and functional properties such as texture, sensory attributes, physical and chemical stability during storage. Generally, food processing operations affect food microstructure: existing structures are destroyed, and new ones are created. Hence, it is important to study the microstructure of food to form high-quality food (Devahastin, 2017). Commonly, the study of the microstructure is conducted by 2D techniques like optical microscopy and electron microscopy: Scanning electron microscopy and Transmission Electron Microscopy. XRMT is an emerging advanced 3D technique that gives food microstructure by a non-invasive, non-destructive, and minimal sample preparation requirement. This technique is particularly interesting for cellular materials like baked goods due to the high contrast between air voids and the solid phase (Van Dyck et al., 2014). XRMT has been successfully applied on bakery products to understand (a) the evolution of dough during fermentation (Shehzad et al. (2010)), (b) the structure of bread crumb (Falcone et al., 2004)), dough development during proofing and baking (Besbes et al., 2013) (d) bubble size distribution of freeze-dried dough and foam properties of bread, bread porous structure (Falcone et al., 2004; Germishuys & Manley, 2021).

The microstructure of bread depends on physical and chemical changes occurring on flour components (flour, water, salt, yeast, and fat) and different processing stages. Three main processing stages that affect bread's microstructure are mixing, fermentation, and baking. Mixing is crucial in creating and retaining small cylindrical air cells inside the food matrix, which defines its cellular structure. The dough oxidation is enhanced by air incorporated in bread, which aids in forming disulfide bonds and ultimately forming a gluten network. During fermentation, the released carbon dioxide moves towards gas nuclei leading to dough expansion and volume increase in the dough. Finally, the dough is subjected to high temperature during baking, leading to gas expansion and stretching of dough. Expansion of dough during baking is dependent on protein coagulation and starch gelatinization. Water availability plays an essential factor for protein coagulation during baking (Cafarelli et al., 2014a; Devahastin, 2017). Hence, XRMT analysis was conducted to understand whether the application of CP incorporates any changes during the different processing stages that may affect bread microstructure.

**Fig. 5:**
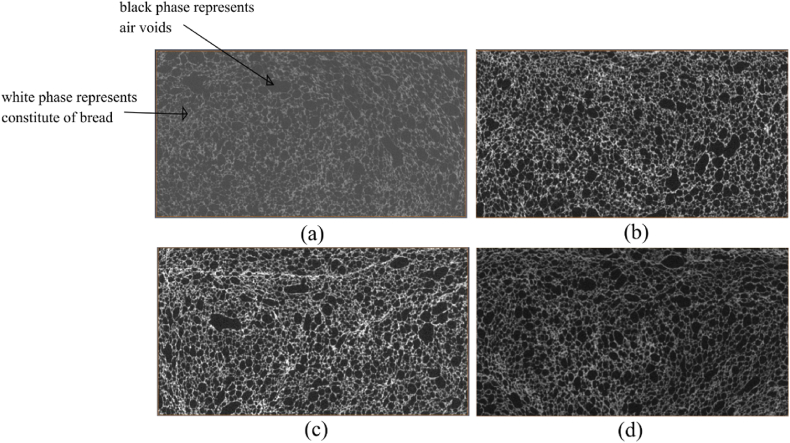
Grayscale images of bread samples where (a) control is bread formulated from untreated flour (b) PFFB-10 is bread formulated from flour treated for 10 min (c) PFFB-20 is bread formulated from flour treated for 20 min (d) PFFB-30 is bread formulated from flour treated for 30 min.

Fig. 5 shows grayscale reconstructed cross-section images of bread from different treatment times. Grayscale images consist of a white and black region formed by variation in absorption of X-rays. The white region shows higher absorption of X-Rays, i.e., high-density region by material, and the black region shows lower absorption, i.e., air voids and gas phase. As seen in Fig. 5, that black region represents air voids, and the white region covers an area for solid-phase distribution (Cafarelli et al., 2014b). It was apparent that all images showed a clear contrast between white and dark spots, showing that CP treatment has not adversely affected the gluten network formation during the mixing stage and the retention of dough structure during baking.

**Fig. 6:**
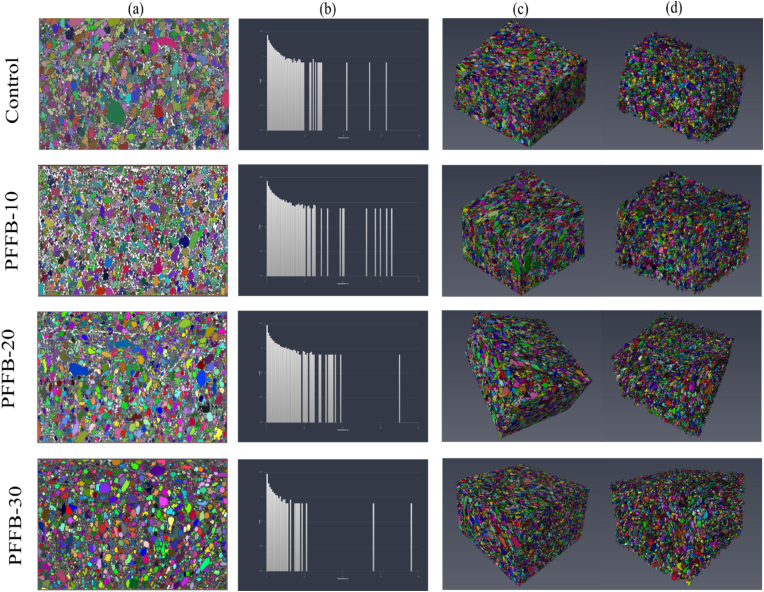
XRMT images of a) central section extracted from each bread with air voids separated and labelled b) histogram of size distribution of air voids c) 3D reconstruction of air voids d) 3-D reconstruction of air voids where, air voids touching the bounding box were removed, voxel size 0.073 × 0.073 × 0.073 mm.

Fig. 6 shows XRMT images of bread samples in 2-D and 3-D reconstructed form. For the analysis of air voids, a central region is extracted from each specimen to eliminate artefacts or any edge damage. Porosity analysis was performed at a constant volume of interest as 195436.686 mm³. Fig. 6a represents the 2-D reconstructed image where air voids have been separated and removed for analysis, Fig. 6b is a graphical representation of the voxel intensity histogram, and Fig. 6c and d are the 3-D representation of the same central extracted region wherein Fig. 6d air voids touching the bounding box have been removed for statistical analysis.

Table 4 overviews 3-D morphological parameters for control and PFFB samples. The air void volume of control was 106237.4141 mm^3,^ and with increasing treatment time, air void volume has declined. The air void volume followed the same trend when the bounding box was removed. The porosity of control bread was 54.44%, and for PFFB-30 was 45.2%. Cafarelli et al., 2014a observed that pore volume and surface increase are independent of the yeast and water content; hence, the addition of extra water (10 g) during the mixing stage for PFFB should not affect its morphological characteristics. Many authors have reported the porosity of various types of bread in the range of 70–80% (Danev et al., 2019). However, porosity depends on the type of bread, processing technique, dough expansion constraint by baking pan, and addition of bran (Van Dyck et al., 2014). Falcone et al. (2004) conducted XRMT analysis to understand the porous structure of white pan and Apulia bread and suggested that porosity alone should not be considered to correlate bread quality; other factors such as cell wall thickness and fractional dimensions should be considered. Overall, PFFB flour bread compared well with control in microstructural analysis.

**Table 4:** 3-D parameters characterizing the morphology of central section extracted from each bread. The control is untreated flour bread, PFFB-10, PFFB-20, and PFFB-30 are bread formulated from flour treated for 10, 20 and 30 min respectively.

Sample	Total extracted central section region volume (mm^3^)	Air voids volume (mm^3^)	Air voids volume (bounding box removed) (mm^3^)	Porosity
**Control**	195436.686	106237.4141	85780.21875	0.544
**PFFB-10**	195436.686	97483.23438	76210.65625	0.499
**PFFB-20**	195436.686	88012.19531	70743.77344	0.450
**PFFB-30**	195436.686	88290.21094	75667.53125	0.452

### 3.7 Texture analysis

The textural properties of bread were calculated from the force-time graph using texture profile analysis. Significant variations in the hardness value for control bread (6.794 ± 0.45 N) and PFFB-30 bread (7.85 ± 0.3 N) were noted. The hardness value of pan bread with traditional strain was 736 g (Nassar et al., 2017). Though the application of plasma treatment increased the force required to compress the bread, the result is in range of literature cited by other authors untreated pan bread. In addition, the XRMT analysis result suggested a decrease in porosity with plasma treatment, making denser gluten. The increased density of the gluten network provided more resistance to compression than the control (Bhise & Kaur, 2014). Misra et al. (2015) also observed an improvement in dough strength for both weak and hard flour with DBD plasma treatment. An increase in disulfide bonds was observed with the treatment, leading to a denser bread crumb. The main parameters affecting the bread texture profile are water activity, acidity values, and gluten quality (Armero & Collar, 1997; Patrignani et al., 2014). In earlier studies, a decrease in α-amylase activity and increase in gluten content following ozone treatment has been associated with the prevention of transfer of insoluble starch to soluble starch, thus making a stronger dough with an increased hardness value of steamed wheat bread (Mei et al., 2016). A similar increase in hardness value was observed with the application of HPP on wheat bread (Bárcenas et al., 2010).

### 3.8 Sensory analysis

**Fig. 7:**
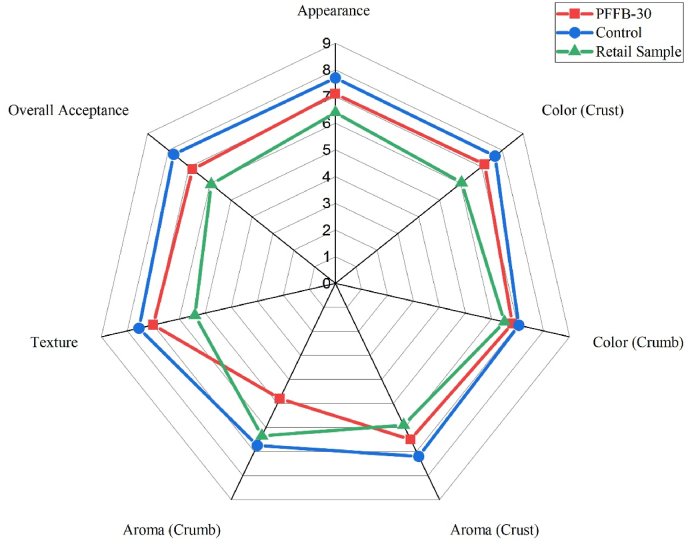
Sensory analysis profile of PFFB-30, control and retail sample. The parameters considered were crust color, crumb color, texture, aroma (crumb), aroma (crust), appearance and overall acceptance. (For interpretation of the references to color in this figure legend, the reader is referred to the Web version of this article.)

Fig. 7 represents a radar chart for sensory analysis of three samples: control bread, PFFB, and retail samples (purchased from SuperValu). The PFFB bread was compared with the in-house control and retail sample of similar dimensions to remove any bias due to the difference in size. The overall acceptance for control, PFFB-30, and the retail sample were 7.75 ± 0.82, 6.85 ± 1.35, and 6.25 ± 1.44, respectively. The overall acceptance of control was rated highest, but the PFFB-30 value was not significantly different compared to both control and retail samples (p < 0.05). The retail sample rated low (6.05 ± 1.56) on the crust color due to the crust's darker color compared to the other two samples. The appearance and crust color are the primary factors in purchasing bread products for the consumers; a mixed review for crust color was observed among panelists. Some panelists preferred the dark color crust, relating it to be properly baked, while others related the dark crust color as overbaked or burned. The color analysis result suggested an increase in the L* value of bread crust due to plasma treatment; however, the overall acceptance of PFFB-30 and control were similar (p < 0.05). Further images of the bread samples are within supplementary images.

While the porosity of the bread decreased with the application of cold plasma treatment compared to control, texture (by touch) did not show a significant difference in the PFFB-30, control, and market sample 7.00 ± 1.18, 7.55 ± 0.86, and 6.40 ± 1.59. Visually, the variation in the porosity did not affect the panelist's perception of the bread crumb texture. The improvement in the water activity of bread crumb can indicate that the panelist did not find the bread to be denser. The PFFB-30 rated lowest on the aroma of crumb (4.8 ± 1.96) compared to others; very few panelists preferred the smell of bread, while others did not favor it. The possible reason for PFFB-30 aroma can be the smell of Vitamin B3 (Niacin) which was a part of the composition of flour. Niacin is a derivative of pyridine, which has an unpleasant fish-like smell (Scriven & Murugan, 2005). A 20% increase in niacin concentration of siriguela juice after 10 min of plasma treatment was noted by (Paixão et al., 2019). In our study, the plasma treatment may have increased niacin's accessibility, which resulted in the unacceptable aroma. For the texture attribute, all samples scored well, with no significant difference recorded (p < 0.05). Overall, plasma-treated flour scored well compared to both control and retail samples.

The literature on sensory analysis of non-thermally treated flour bread is scarce. The sensory evaluation of bread formed from the HPP treated dough (50–250 MPa) had consumer acceptance up for 50 and 100 MPa treated dough only. The HPP treatment altered the textural properties of bread, giving a croissant-like texture (Bárcenas et al., 2010). Singer et al. (2006) conducted a triangle sensory test on irradiated flour bread (1.0 kGy and 2.0 kGy), and non-irradiated bread and panelist observed no difference in acceptance of both breads. At higher irradiation doses (4.0 kGy and 8.0 kGy), consumer acceptance of irradiated flour bread was decreased (Zaied et al., 1996). The further development of non-thermal technologies such as cold plasma for treatment of grain and flour and the products developed from these warrants further focus on all sensory effects and what reactions these may be attributed to, to understand how the product development process can be adapted to exploit the techno-functional advantages of cold plasma processing of flour but also to understand what the limitations are.

## 4 Conclusion

Plasma functionalized ingredients can be exploited to form cereal-based products. In our study, pan bread was developed from plasma functionalized flour treated for 5–30 min at 80 kV air plasma. The expansion ratio of PFFB was greater than that of control, making the bread rise higher during baking. The results further revealed that the water activity of PFFB crumb was higher and color analysis suggested a decrease in L* value compared to control. It should be noted that PFFB scored well among different sensory attributes with no significant difference in overall acceptability when compared to the control and retail samples. The farinograph characteristics of plasma-treated wheat flour dough provided useful information on modifying the effect of plasma treatment on the behaviour of the dough, indicating an improvement in dough properties like water absorption capacity, dough development time, and stability time.

XRMT is a non-destructive analysis of the 3D microstructure of food matrix and their interaction with processing parameters in such a way as to allow not only a fundamental understanding of the process but also a process design resulting in specific desired microstructures. In general, the XRMT images of PFFB demonstrated no change in the gluten network and an increased porosity by comparison with control. Thus, cold plasma treatment did not adversely affect the dough structure formation. Cold plasma has been studied previously to enhance the functionality of a different variety of wheat flour; our present work further confirms that a new product with improved properties can be developed from plasma functionalized flour. Thus, cold plasma can be exploited as a more sustainable alternative to current FDA-approved additives to improve dough properties and, subsequent to thorough sensory testing, can prove a useful tool in new product development and new ingredient formulations.

## CRediT authorship contribution statement

**Sonal Chaple:** conceived the work, Formal analysis, Writing – review & editing, wrote the manuscript, reviewed and edited the manuscript. **Chaitanya Sarangapani:** Formal analysis, Writing – review & editing, conceived the work, analyzed data, wrote the manuscript, reviewed and edited the manuscript. **Shannon Dickson:** Formal analysis, Writing – review & editing, conceived the work, analyzed data, wrote the manuscript, reviewed and edited the manuscript. **Paula Bourke:** Writing – review & editing, conceived the work, wrote the manuscript, reviewed and edited the manuscript.

## Declaration of competing interest

The authors declare that they have no known competing financial interests or personal relationships that could have appeared to influence the work reported in this paper.
