# Supplementary material for: Product development and X-Ray microtomography of a traditional white pan bread from plasma functionalized flour
Source: Lebensm Wiss Technol. 2023 Jan 15;174:114326. doi: 10.1016/j.lwt.2022.114326 (PMC9883616; doi:10.1016/j.lwt.2022.114326)
Supplement: Multimedia component 1 [file mmc1.docx]

**Product development and X-Ray microtomography of a traditional white pan bread from plasma functionalised flour**

Sonal Chaple^1^, Chaitanya Sarangapani^2^, Shannon Dickson^3^, Paula Bourke* ^1^

^1^School of Biosystems and Food Engineering, University College Dublin, Belfield, Dublin 4, Ireland

^2^School of Food Science and Environmental Health, Technological University Dublin, Dublin 7, Ireland

^3^School of Culinary Arts and Food Technology, Technological University Dublin, Dublin 7, Ireland


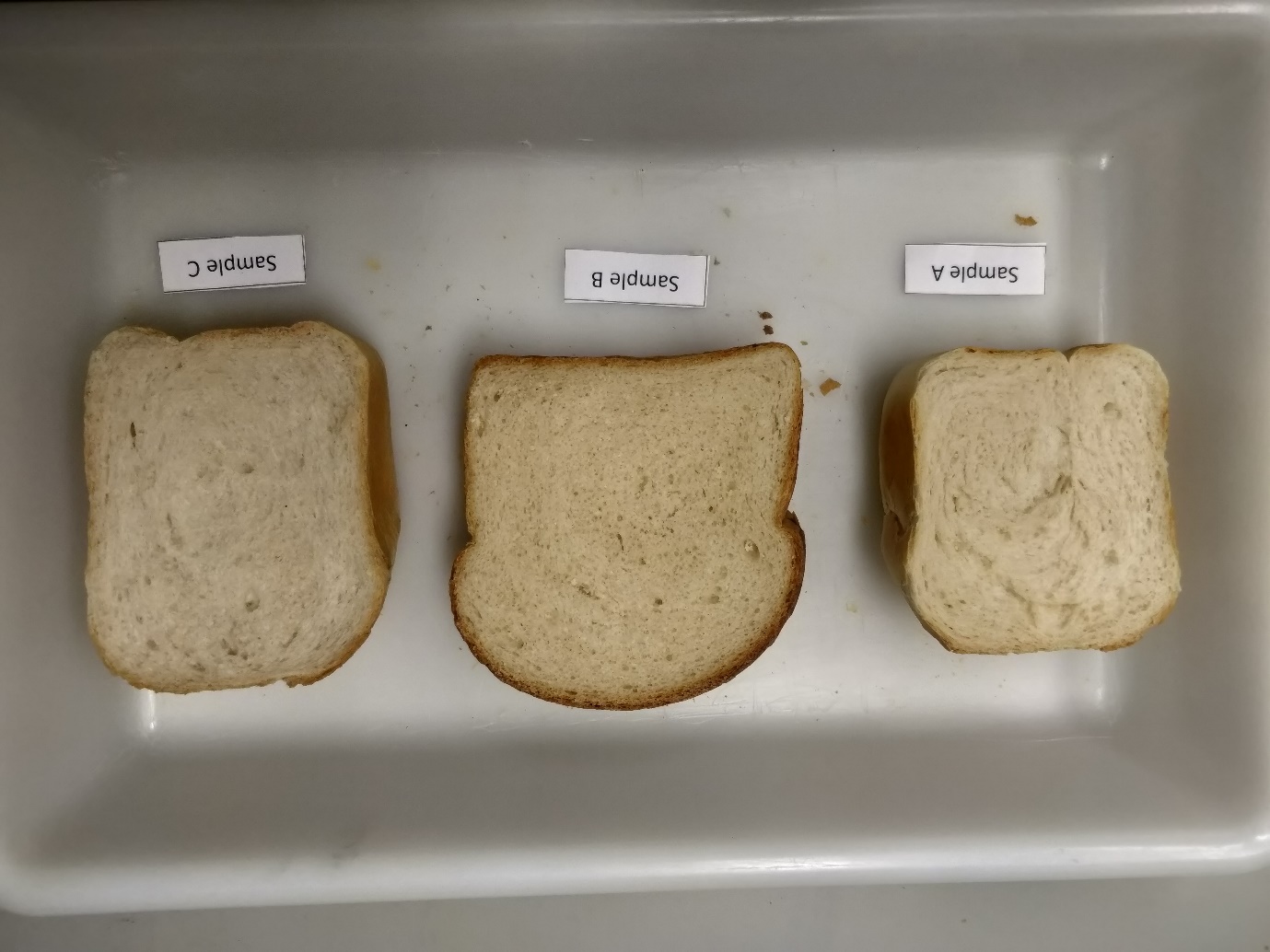
*Corresponding author: paula.bourke@ucd.ie

**Supplementary image**

**Fig A:** Sensory analysis set up consumer acceptance test. Where Sample A is control bread, Sample B is market sample and Sample C is PFFB-30 bread

**Table. A.** Water activity of bread crust and crumb. The control is untreated flour bread, PFFB-10, PFFB-20 and PFFB-30 are bread formulated from flour treated for 10, 20 and 30 min respectively. All the data are expressed as mean± standard deviations. Means with different superscript letters in a column differ significantly (*p*<0.05)

| Sample | Bread Crust | Bread Crumb |
| --- | --- | --- |
| Control | 0.904±0.02^a^ | 0.958±0.008^a^ |
| PFFB-10 | 0.913±0.01^ab^ | 0.963±0.003^ab^ |
| PFFB-20 | 0.92±0.02^b^ | 0.972±0.004^b^ |
| PFFB-30 | 0.937±0.02^c^ | 0.986±0.001^c^ |

**Table. B.** Sensory analysis table for control, market sample and PFFB-30 (bread formulated from flour treated for 30 min). All the data are expressed as mean± standard deviations. Means with different superscript letters in a column differ significantly (*p*<0.05)

| Sensory Attributes | PFFB-30 | Control | Market sample |
| --- | --- | --- | --- |
| Appearance | 7.10±1.45^a^ | 7.70±0.71^ab^ | 6.40±1.77^b^ |
| Crust (Color) | 7.15±1.53^a^ | 7.65±1.11^ab^ | 6.05±1.56^b^ |
| Crumb (Color) | 6.80±1.08^a^ | 7.05±1.20^a^ | 6.50±1.69^a^ |
| Aroma (Crust) | 6.50±2.25^a^ | 7.20±1.33^a^ | 5.90±1.64^a^ |
| Aroma (Crumb) | 4.80±1.96^a^ | 6.75±1.67^b^ | 6.35±1.49^b^ |
| Texture (By fingers) | 7.00±1.18^a^ | 7.55±0.86^a^ | 6.40±1.59^a^ |
| Overall acceptance | 6.85±1.35^a^ | 7.75±0.83^a^ | 6.25±1.75^a^ |
